# Cannabidiol negatively modulates adenosine A_2A_ receptor functioning in living cells

**DOI:** 10.1017/neu.2023.30

**Published:** 2023-08-22

**Authors:** Nuria Sánchez-Fernández, Laura Gómez-Acero, Laura I. Sarasola, Josep Argerich, Andy Chevigné, Kenneth A. Jacobson, Francisco Ciruela, Víctor Fernández-Dueñas, Ester Aso

**Affiliations:** 1 Pharmacology Unit, Department of Pathology and Experimental Therapeutics, School of Medicine and Health Sciences, Institute of Neurosciences, University of Barcelona, L’Hospitalet de Llobregat, Spain;; 2 Neuropharmacology and Pain Group, Neuroscience Program, Institut d’Investigació Biomèdica de Bellvitge, IDIBELL, L’Hospitalet de Llobregat, Spain;; 3 Immuno-Pharmacology and Interactomics, Department of Infection and Immunity, Luxembourg Institute of Health (LIH), Esch-sur-Alzette, Luxembourg; 4 Molecular Recognition Section, Laboratory of Bioorganic Chemistry, National Institute of Diabetes and Digestive and Kidney Diseases, National Institutes of Health, Bethesda, MD, USA

**Keywords:** cannabidiol, adenosine 2A receptor, negative allosteric regulation, competitive binding, cyclic AMP, luminescence-based assays

## Abstract

**Objectives:**

Cannabidiol (CBD) is a phytocannabinoid with great potential in clinical applications. The mechanism(s) of action of CBD require further investigation. Previous studies suggested that adenosine A_2A_ receptors (A_2A_Rs) could play a role in CBD-induced effects. Here, we evaluated the ability of CBD to modify the function of A_2A_R.

**Methods:**

We used HEK-293T cells transfected with the cDNA encoding the human A_2A_R and Gαs protein, both modified to perform bioluminescence-based assays. We first assessed the effect of CBD on A_2A_R ligand binding using an A_2A_R NanoLuciferase sensor. Next, we evaluated whether CBD modified A_2A_R coupling to mini-Gαs proteins using the NanoBiT^™^ assay. Finally, we further assessed CBD effects on A_2A_R intrinsic activity by recording agonist-induced cAMP accumulation.

**Results:**

CBD did not bind orthosterically to A_2A_R but reduced the coupling of A_2A_R to Gαs protein and the subsequent generation of cAMP.

**Conclusion:**

CBD negatively modulates A_2A_R functioning.

## Introduction

Cannabidiol (CBD) is a phytocannabinoid isolated from *Cannabis sativa* without psychoactive properties, but with potential benefits against multiple pathological conditions (ElSohly *et al*., 2017). Several preclinical reports demonstrated protective and anti-inflammatory effects of CBD in a wide spectrum of neurodegenerative diseases, neuroinflammatory processes, stroke, colitis, liver, kidney injury, cardiovascular disease, arthritis, sepsis, diabetes, cancer, and epilepsy models (Pacher *et al*., 2020). Furthermore, CBD exerted positive effects in experimental models of other neuropsychiatric disorders such as epilepsy, anxiety, schizophrenia, dementia, addiction, and neonatal hypoxic-ischemic encephalopathy (Devinsky *et al*., 2014). Although its translation to clinical trials is somewhat limited to date, the successful case of Epidiolex^®^, an oral solution based on a botanical extract containing purified CBD, is notable. Epidiolex^®^ was approved by the US Food and Drug Administration in 2018 for the treatment of Lennox-Gastaut and Dravet syndromes, two rare and debilitating genetic forms of epilepsy in children. Additionally, CBD is currently under clinical evaluation for other conditions, including different forms of pain, obsessive-compulsive disorders, and behavioural problems associated with intellectual disability or autism, among others (ClinicalTrials.gov database).

Despite growing interest in its potential clinical applications, the mechanism(s) of action of CBD require further exploration. CBD has a very low affinity for the orthosteric site of CB_1_ and CB_2_ receptors, the main G protein-coupled receptors (GPCRs) that belong to the endogenous cannabinoid system (McPartland *et al*., 2015). Alternatively, CBD can act on multiple targets, including TRPV1 channels and PPARγ, adenosine A_2A_, 5-HT_1A_, α_3_-glycine, α_1_-adrenal, dopamine D_2_, GABA_A_, μ- and δ-opioid receptors (McPartland *et al*., 2015). Additionally, CBD can inhibit the activity of GPR55 (Ryberg *et al*., 2007), an effect that has been associated with its antiepileptic activity (Sylantyev *et al*., 2013). In the present study, we probed the putative direct effects of CBD on adenosine A_2A_ receptors (A_2A_Rs). The relevant role that A_2A_Rs play in several of the neuropsychiatric disorders in which CBD could offer beneficial effects (i.e. dementia, schizophrenia, epilepsy, depression, anxiety) supports this interest (Domenici *et al*., 2019). Furthermore, previous preclinical evidence supports the participation of A_2A_R in CBD-mediated effects. Thus, A_2A_R antagonists blocked the anti-inflammatory effects of CBD (Liou *et al*., 2008; Ribeiro *et al*., 2012; Mecha *et al*., 2013; Oláh *et al*., 2014), or the ability of CBD to blunt Δ^9^-THC-induced cognitive impairment (Aso *et al*., 2019). Similarly, the genetic deletion of A_2A_R reduced the CBD-induced potentiation of the cataleptic and anxiolytic properties of Δ^9^-THC (Stollenwerk *et al*., 2021). This A_2A_R-dependent activity of CBD was proposed to depend on the ability of CBD to bind to the equilibrative nucleoside transporter (ENT). Thus, inhibition of adenosine uptake would lead to indirect activation of A_2A_R (Pandolfo *et al*., 2011). However, a direct effect of CBD on A_2A_R has not been further investigated. Here we aimed to evaluate the capacity of CBD to bind to the orthosteric site of A_2A_R and/or to modify its intrinsic activity by using state-of-the-art luminescence-based assays.

## Materials and methods

### Reagents

The ligands used were CGS21680, ZM241385, and CBD (Tocris Bioscience, Bristol, United Kingdom). MRS7396, a fluorescent selective A_2A_R orthosteric antagonist derived from SCH442416 and containing a BODIPY630/650 fluorophore, was previously described (Duroux *et al*., 2017). Other reagents used were Dulbecco’s modified Eagle’s medium (DMEM; Sigma Aldrich, St Louis, MO, USA), geneticin (Santa Cruz Biotechnology, Dallas, TX, USA), adenosine deaminase (ADA; Roche Diagnostics GmbH, Mannheim, Germany), zardaverine (Calbiochem, San Diego, CA, USA), and coelenterazine 400a (NanoLight Technologies, Pinetop, AZ, USA).

### Plasmid constructs

To perform bioluminescence resonance energy transfer (BRET) experiments and cAMP accumulation assays, we used the A_2A_R NanoLuciferase (NanoLuc) sensor (A_2A_R^NL^), previously described (Lanznaster *et al*., 2019). To perform the NanoBiT^™^ assay, the cDNA encoding human A_2A_R was cloned at the BamHI/EcoRV restriction enzyme sites of pIREShyg3-SmBiT (Promega, Madison, WI, USA), as previously described (Sarasola *et al*., 2022). The construct (A_2A_R^SmBiT^) was verified by DNA sequencing. The plasmid encoding the mini-Gαs (engineered GTPase domain of Gα subunit; LgBiTmini-Gαs) linked to LgBiT was previously described (Wan *et al*., 2018; Meyrath *et al*., 2021).

### Cell culture and transfection

Human embryonic kidney (HEK)-293T cells were grown in DMEM supplemented with 1 mM sodium pyruvate (Biowest, Nuaillé, France), 2 mM L-glutamine (Biowest), 100 U/mL streptomycin (Biowest), 100 mg/mL penicillin (Biowest), and 5% (*v*/*v*) foetal bovine serum (Invitrogen Corporation, Camarillo, CA, USA) at 37°C and in an atmosphere of 5% CO_2_. Cells were transiently transfected with the indicated cDNA construct using polyethylenimine (PEI, 1 mg/mL, Sigma Aldrich), as previously described (Longo *et al*., 2013). Finally, HEK-293T cells stably expressing A_2A_R^NL^ were grown in the presence of geneticin (1 mg/mL).

### NanoBRET experiments

The NanoBRET assay was performed as previously described (Lanznaster *et al*., 2019). Briefly, HEK-293T cells expressing the A_2A_R^NL^ construct were resuspended in Hank’s balanced salt solution (HBSS; Thermo Fisher, Waltham, MA, USA) containing ADA (0.5 U/mL) and plated on white 96-well plates coated with poly-ornithine (Corning, Corning, NY, USA) at a density of 20,000 cells/well. After 24 h, cells were challenged with the fluorescent A_2A_R antagonist (MRS7396) in the absence/presence of ZM241385 or CBD and incubated for 1 h at 37°C. Subsequently, coelenterazine 400a was added at a final concentration of 1 μM, and the readings were performed after 15 min using a CLARIOStar microplate reader (BMG Labtech, Durham, NC, USA). Donor and acceptor emission were measured at 490 ± 10 nm and 650 ± 40 nm, respectively. The raw NanoBRET ratio was calculated by dividing the 650 nm emission by the 490 nm emission and the values fitted by nonlinear regression using GraphPad Prism 9 (GraphPad Software, La Jolla, CA, USA). The results were expressed as a percentage of the maximum signal obtained (mBU; miliBRET units).

### NanoBiT assay

The NanoBiT^™^ assay (Promega) was performed as previously described (Sarasola *et al*., 2022). Briefly, transient transfected HEK-293T cells with A_2A_R^SmBiT^ and ^LgBiT^mini-Gαs were resuspended in HBSS containing ADA (0.5 U/mL) and transferred (90 μl) into white 96-well plates (Corning). Subsequently, coelenterazine 400a was added (1 μM) to each well. After 15-minute incubation, basal luminescence was determined using a CLARIOstar plate reader (BMG Labtech). Immediately after the initial measurement (basal), the ligands were added, and the luminescent signal was measured every 5 min for 30 min. The luminescence signal (RLU) was normalised as follows: (RLU_sample_ – RLU_basal_) / RLU_basal_.

### cAMP assay

cAMP accumulation was measured using the LANCE^®^ Ultra cAMP Kit (PerkinElmer, Waltham, MA, USA) as previously described (Lanznaster *et al*., 2019). Briefly, HEK-293T cells stably expressing the A_2A_R^NL^ construct were first incubated for 1 h at 37°C with stimulation buffer (BSA 0.1%, ADA 0.5 units/mL, zardaverine 2 μM; in serum-free DMEM) and later with CGS21680 (100 nM) and increasing concentrations of ZM241385 or CBD for 30 min at 37°C. Subsequently, cells were transferred (1000 cells/well) into white 384-well plates (Corning), in which reagents were added following the manufacturer’s instructions. After 1 h at room temperature, time-resolved fluorescence resonance energy transfer (TR-FRET) was determined by measuring light emission at 620 nm and 665 nm using a CLARIOstar plate reader (BMG Labtech).

### Statistics

Data are represented as mean ± standard error of mean (SEM) with statistical significance set at *P* < 0.05. The number of samples (n) in each experimental condition is indicated in the legend of the corresponding figure. Outliers were assessed using the ROUT method (Motulsky & Brown, 2006); thus, no sample was excluded assuming a *Q* value of 1% in GraphPad Prism 9. Comparisons between experimental groups were performed using one-way factor analysis of variance (ANOVA) followed by Dunnett’s multiple comparisons *post hoc* test using GraphPad Prism 9 as indicated.

## Results

**Figure 1.**
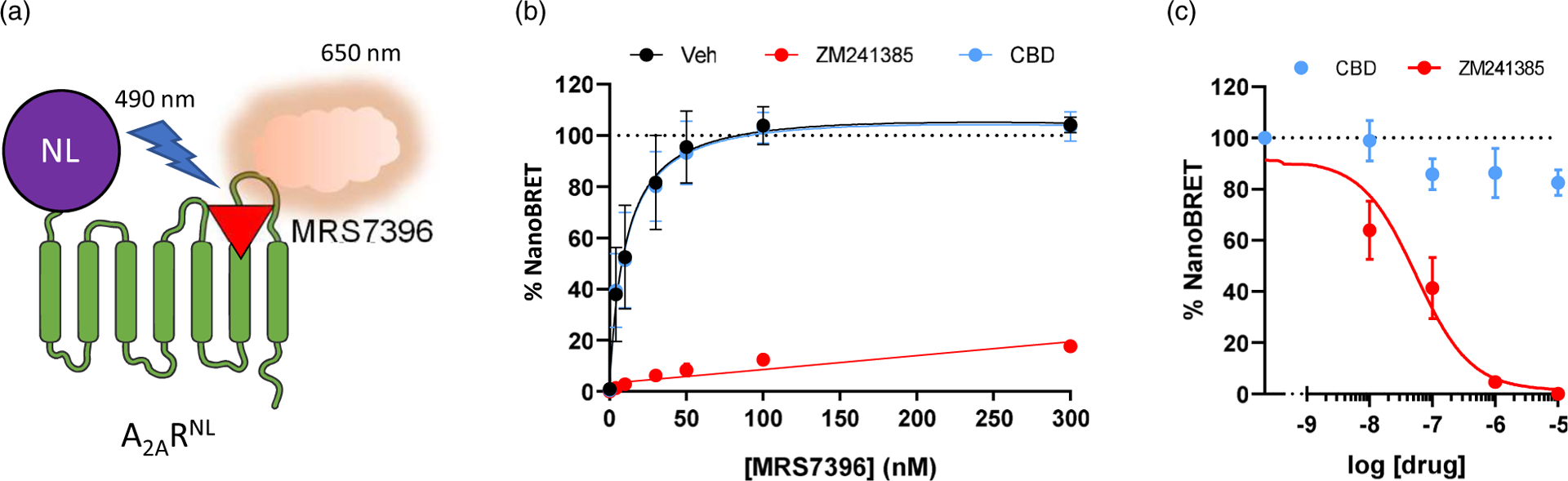
Determination of CBD effects on A_2A_R ligand binding affinity**. (a)** Schematic representation of the NanoBRET assay. A nanoluciferase is linked to the N-terminal part of the A_2A_R (A_2A_R^NL^). When the nanoluciferase substrate coelenterazine is added, A_2A_R^NL^ (donor) emits light at 490–10 nm. Light excites the fluorescent selective A_2A_R ligand, MRS7396 (acceptor), which subsequently emits fluorescence at 650–80 nm. **(b)** NanoBRET saturation binding curves obtained by challenging A_2A_R^NL^ expressing HEK-293T cells with increasing concentrations of MRS7396 in the absence/presence of CBD (1 μM) or ZM241385 (1 μM). **(c)** NanoBRET signals obtained by challenging A_2A_R^NL^ expressing HEK-293T cells with a fixed concentration of MRS7396 (30 nM, normalised to 100%) in the presence of increasing concentrations of CBD or ZM241385. The represented data are mean ± SEM of three independent experiments each performed in triplicate.

To assess the impact of CBD on A_2A_R functionality, we initially evaluated whether CBD modified the binding of MRS7396, a fluorescent A_2A_R antagonist. To this end, we took advantage of a previously reported NanoBRET-based A_2A_R binding assay (Fig. 1a) (Lanznaster *et al*., 2019). HEK-293T cells permanently expressing the A_2A_R^NL^ construct were challenged with increasing concentrations of MRS7396, which upon binding to the receptor can act as a compatible acceptor in a BRET process (Fig. 1a). As expected, a saturable hyperbolic curve was obtained for the total binding of MRS7396, which was blocked upon incubation with a saturating concentration (1 μM) of the unlabelled A_2A_R antagonist ZM241385 (nonspecific binding; Fig. 1b). The analysis of the specific binding of MRS7396 revealed a dissociation constant (*K*_D_) of 8.5 ± 3.2 nM and a maximum binding capacity (*B*_max_) of 98.9 ± 8.4 %. Next, upon the same experimental conditions, we examined the ability of CBD to attenuate MRS7396 binding. Differently from ZM241385, CBD (1 μM) was unable to modify the specific binding of MRS7396 to the A_2A_R (Fig. 1b). Accordingly, no significant differences in affinity (*K*_D_) and receptor capacity (*B*_max_) were found in the presence of CBD (K_D_ = 8.2 ± 2.7 nM; B_max_ = 96.9 ± 7.2 %; *P* = 0.992, *F*_(2, 34)_ = 0.0079). In addition, we also assessed whether CBD could modulate orthosteric binding of A_2A_R by performing a competition binding assay with a fixed concentration of MRS7396 (30 nM). Again, while ZM241385 blocked A_2A_R binding, CBD did not significantly modify the NanoBRET signal (Fig. 1c; *P* = 0.269, *F*_(4, 10)_ = 1.518). Collectively, these results indicate that CBD does not bind orthosterically to A_2A_R.

**Figure 2.**
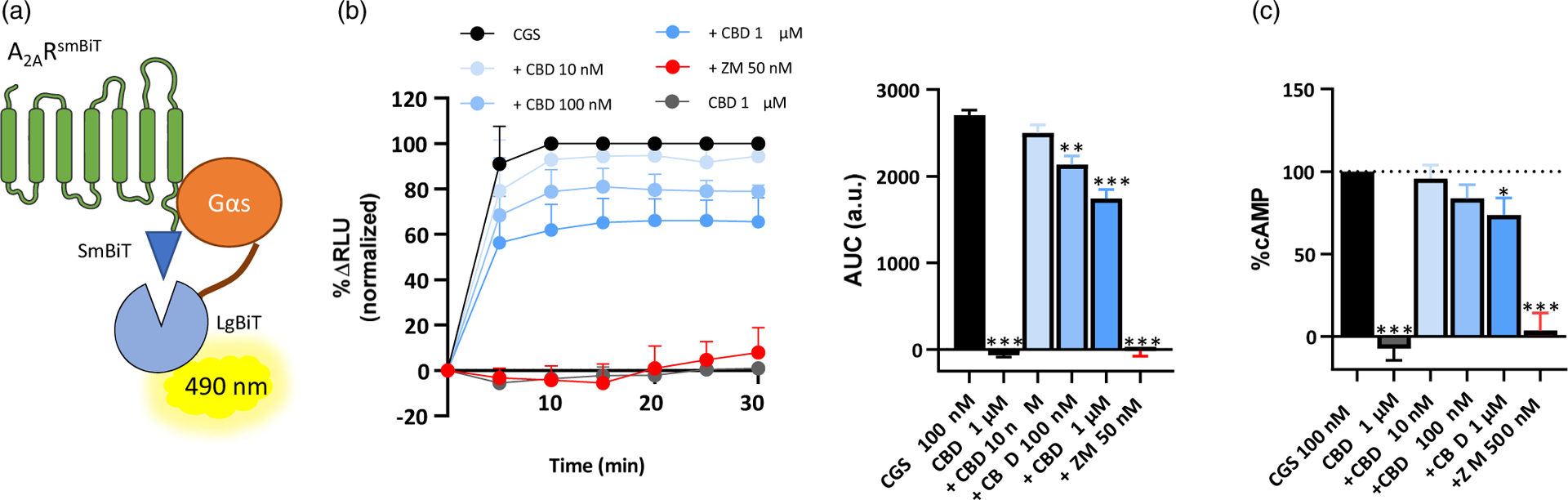
Assessment of CBD effects on A_2A_R intrinsic activity**. (a)** Schematic representation of the NanoBIT^™^-based assay. The two fragments of nanoluciferase, small (SmBIT) and large (LgBIT), are fused to A_2A_R and mini-Gαs protein, respectively. Then, upon agonist binding, A_2A_R intrinsic activity is assessed by receptor recruitment of Gαs, which induces an increase on luminescence due to nanoluciferase reconstitution. **(b)** Representative time-course of A_2A_R agonist-mediated Gαs recruitment. The selective A_2A_R agonist CGS21680 was challenged to A_2A_R^SmBiT^ and ^LgBiT^ mini-Gαs expressing HEK-293T cells in the absence/presence of increasing concentrations of CBD or ZM241385. The luminescent signal obtained after reconstitution of the nanoluciferase was assessed by calculating the area under the curve for each condition. Data are shown as mean ± SEM of three independent experiments with five replicates. *P < 0.05, ***P < 0.001, one-way ANOVA with Dunnett’s *post hoc* test. **(c)** cAMP accumulation was assessed on HEK-293T cells permanently expressing the A_2A_R^NL^. Cells were challenged with the selective A_2A_R agonist CGS21680 (100 nM, normalised to 100% of effect) in the absence/presence of increasing concentrations of CBD. Data are expressed as mean ± SEM of four independent experiments performed in triplicates. *P < 0.05, one-way ANOVA with Dunnett’s *post hoc* test.

Subsequently, we aimed to determine whether CBD affected A_2A_R signalling. To this end, we first evaluated the interaction of A_2A_R with mini-Gαs protein using the NanoBiT^™^ complementation assay (Sarasola *et al*., 2022). Accordingly, cells were transiently transfected with the A_2A_R^SmBiT^ and ^LgBiT^mini-Gαs constructs, which once expressed allow the reconstitution of the split nanoluciferase and real-time recordings of receptor-effector coupling induced by agonists (Fig. 2a). Cells were challenged with the selective A_2A_R agonist CGS21680 (100 nM), which induced a rapid increase in the luminescent signal, reaching a peak at 15 min that remained stable for 30 min. This effect was absent in cells challenged with CBD alone (Fig. 2b). Notably, this agonist-dependent A_2A_R interaction with mini-Gαs protein was completely blocked when co-incubating cells with ZM241385 (50 nM, Fig. 2b). Next, we assessed CGS21680-induced A_2A_R coupling to Gαs protein in the presence of increasing concentrations of CBD. Interestingly, CBD dose-dependently blocked A_2A_R coupling to Gαs protein, both decreasing the maximum peak and the density of the effect (Fig. 2b). Of note, differently from ZM241385, CBD only led to a partial blockade of CGS21680-mediated effects.

Finally, to further characterise the effects of CBD on the intrinsic activity of A_2A_R, we evaluated agonist-induced cAMP accumulation in cells permanently expressing the A_2A_R^NL^ construct and challenged with CGS21680 in the presence/absence of CBD. Interestingly, although CBD itself did not modify cAMP levels, it was able to dose-dependently block CGS21680-induced cAMP levels (Fig. 2c). Again, differently from ZM241385, a full-antagonist, CBD partially blocked A_2A_R agonist increase of cAMP levels. Overall, these results are compatible with the notion that CBD acts as a weak A_2A_R negative allosteric modulator.

## Discussion

CBD is a promising drug for several pathologies (ElSohly *et al*., 2017). Accordingly, unravelling its precise mechanism of action is relevant in the progress towards its clinical development. Here, we reveal that CBD does not affect the binding of an A_2A_R orthosteric ligand, but it is capable of negatively modulating agonist-induced interaction with the Gαs protein at sub-micromolar concentrations (≥100 nM), thus reducing receptor signalling (i.e. cAMP generation). Therefore, we disclose a new non-competitive interaction of CBD with A_2A_R.

The effect of CBD on A_2A_R could operate through a new allosteric site at the receptor. However, further experiments (i.e. using labelled CBD) would be needed to confirm this hypothesis. On the other hand, we cannot rule out other mechanisms of action for CBD different from classical allosteric drugs. In this sense, previous evidence indicates that other lipids, including the endogenous cannabinoid anandamide at micromolar concentrations, might act as allosteric modulators of other GPCRs through a membrane-perturbing effect that is sensitive to receptor conformation (Lanzafame *et al*., 2004; Van der Westhuizen *et al*., 2015). Further studies are needed to assess this putative CBD-mediated membrane effect on A_2A_R-Gαs protein coupling. Similarly, CBD could indirectly modify A_2A_R functioning by interacting with equilibrative nucleoside transporter 1 (ENT1), as was previously demonstrated in striatal synaptosomes (Pandolfo *et al*., 2011). However, this hypothetical CBD effect on ENT seems not to play a relevant role *in vivo*, since a recent study demonstrated that CBD lacks the ability to substantially raise endogenous adenosine levels by using the hypothermia mouse model (Xiao *et al*., 2023). These discrepancies between *in vitro* and *in vivo* studies could be also explained by the fact that A_2A_R can form heteromers with other GPCRs, including CB_1_R (Carriba *et al*., 2007; Ferré *et al*., 2010; Aso *et al*., 2019), in physiological conditions different from that obtained in heterologous expression systems. The assembly of A_2A_R-containing heteromers leads to changes in the agonist recognition, signalling, and trafficking, which might result in different A_2A_R activity in the presence of CBD.

Although we evaluated the effects of CBD in cultured cells expressing A_2A_R, these results could be relevant for many disorders in which A_2A_R activity increases. For example, in certain inflammatory processes and cardiovascular diseases, but also in pathological conditions that affect the central nervous system, such as Alzheimer’s disease, Parkinson’s disease, attention deficit hyperactivity disorder, fragile X syndrome, depression, or anxiety (Domenici *et al*., 2019). A_2A_Rs, which are widely expressed both in neurons and glia, are mainly found in the dorsal and ventral striatum and other nuclei of the basal ganglia, where they play a key role in the control of voluntary movements, as well as in motivational, emotional and cognitive processes (Sebastião and Ribeiro, 2009). In this way, A_2A_Rs are involved in regulating the release of neurotransmitters and contribute to the homeostatic control of synaptic transmission and brain function (Sebastião and Ribeiro, 2009). In general, our results are consistent with the positive effects reported for CBD in various brain disorders that can be associated with an exacerbated A_2A_R function, where CBD would tone down A_2A_R hyperactivity.

Overall, the present study provides evidence on the ability of CBD to negatively modulate A_2A_R signalling. The CBD-mediated negative modulation of A_2A_R function is restricted to the receptor-effector coupling and does not interfere with the binding of the orthosteric ligand. Accordingly, we provide a new and genuine pharmacological way to modulate the adenosinergic system in pathological conditions in which A_2A_R function is increased.

## Significant outcomes

- Cannabidiol does not bind orthosterically to A_2A_R.
- Cannabidiol reduces the functionality of A_2A_R

## Limitations

- This is an *in vitro* study, and results cannot be directly extrapolated to *in vivo* conditions.
- Putative allosteric binding of CBD to A_2A_R cannot be confirmed or ruled out with the luminescence-based techniques employed in this study.
